# Think SCN5A: A novel variant causing multifocal Purkinje PVCs and dilated cardiomyopathy

**DOI:** 10.1016/j.ipej.2026.02.003

**Published:** 2026-02-05

**Authors:** Daniel B. Hanna, Andrew Kossack, Juan Perez-Hernandez, Rachael Venn, Luis Rechani, Samantha Sublette, Viviana Navas, Dinesh Sharma

**Affiliations:** Division of Cardiology, Rooney Heart Institute at NCH Healthcare System, Naples, FL, USA

**Keywords:** SCN5A, Multifocal ectopic Purkinje-related premature contractions (MEPPC), Premature ventricular contractions, Purkinje system, Dilated cardiomyopathy, PVC-induced cardiomyopathy, Catheter ablation

## Abstract

**Background:**

Multifocal ectopic Purkinje-related premature contractions (MEPPC) represent a rare *SCN5A*-associated channelopathy characterized by high-burden, multifocal premature ventricular contractions (PVCs) arising from the His–Purkinje system, often leading to reversible left ventricular dysfunction.

**Case summary:**

We describe a young male with a MEPPC-like phenotype and a novel *SCN5A* (c.655G > A, p.Arg222Gln) variant presenting with multifocal Purkinje and papillary muscle PVCs and left ventricular systolic dysfunction. Sequential focal ablations reduced the dominant ectopic burden, while adjunctive flecainide therapy further suppressed residual Purkinje activity and improved ventricular function.

**Conclusion:**

In young patients with multifocal Purkinje PVCs and unexplained cardiomyopathy, consider SCN5A mutation. Combined ablation and sodium-channel blockade could be considered as a treatment option.

## 1 Introduction

Multifocal ectopic Purkinje-related premature contractions (MEPPC) syndrome is a rare arrhythmogenic disorder linked to gain-of-function variants in *SCN5A*, the gene encoding the cardiac sodium channel Nav1.5. The condition is characterized by frequent fascicular or Purkinje premature ventricular contractions (PVCs), often multifocal, which can lead to left ventricular (LV) systolic dysfunction or a dilated cardiomyopathy (DCM)-like phenotype [1].

Despite growing recognition of MEPPC, the syndrome is often undiagnosed in clinical practice. Multifocal PVCs in young patients could easily be attributed to idiopathic causes or PVC-induced cardiomyopathy without consideration of underlying channelopathy. This diagnostic gap could delay appropriate genetic testing and targeted therapy.

Affected individuals typically exhibit enhanced Purkinje excitability and respond to sodium channel–blocking agents such as flecainide, quinidine, hydroquinidine, or amiodarone [2]. While several pathogenic *SCN5A* variants have been described in association with MEPPC, the syndrome remains uncommon, and standardized treatment strategies have not been established [[3], [4], [5], [6], [7]].

We report a case of multifocal Purkinje and papillary muscle PVCs associated with a novel *SCN5A* variant (c.655G > A, p.Arg222Gln), presenting with a MEPPC-like phenotype. This case expands the spectrum of *SCN5A*-associated arrhythmogenic disease and underscores the role of combined pharmacologic and ablative therapies in management.

## 2 Case report

### 2.1 Initial presentation

A 25-year-old Caucasian male with a history of dilated cardiomyopathy and heart failure with reduced ejection fraction (HFrEF) was referred for evaluation of frequent premature ventricular contractions (PVCs). He had a longstanding history of PVCs, first identified on abnormal electrocardiogram (ECG) findings in 2018. A Holter monitor in March 2025 demonstrated a PVC burden of 6%, but repeat monitoring in August 2025 revealed a substantial increase to 36%, correlating with worsening symptoms of palpitations and fatigue.

There was no known family history of sudden cardiac death. However, the patient's father had a history of tachyarrhythmias managed with beta blockers, and his maternal grandmother required a pacemaker.

### 2.2 Investigations

Initial transthoracic echocardiography revealed a moderately reduced left ventricular ejection fraction (LVEF) of approximately 40% in the setting of frequent premature ventricular contractions (PVCs). Cardiac magnetic resonance imaging (CMR) demonstrated further decline in systolic function, with an LVEF of approximately 24%, and showed no evidence of infiltrative, inflammatory, or ischemic cardiomyopathy.

**Fig. 1:**
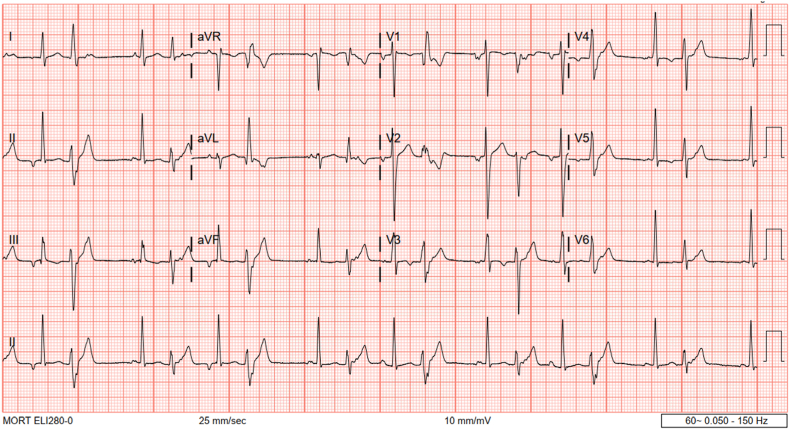
Representative 12-lead electrocardiogram demonstrating sinus rhythm with isolated premature ventricular complexes (PVCs).

The patient was subsequently referred to cardiology service for further evaluation. Extended ambulatory monitoring revealed sinus rhythm with an average heart rate of approximately 100 bpm and a total of 84,000 PVCs, corresponding to a burden of ∼36%. Office ECGs demonstrated sinus tachycardia with frequent multifocal PVCs, narrow QRS width and ST–T wave changes consistent with anterolateral repolarization abnormalities (Fig. 1).

Frequent PVCs, multi-focal Purkinje-origin PVCs and cardiomyopathy raised the suspicion of MEPPC. Genetic testing was ordered and based on patient's preference; catheter ablation was planned.

### 2.3 Procedure

Via transeptal access performed through right femoral vein utilizing an intracardiac echocardiography (ICE) probe (Siemens Medical Solutions, Malvern, PA, USA), left ventricle mapping was performed. Isoproterenol was infused at 1 μg/min to facilitate PVC induction.

The patient exhibited multiple distinct premature ventricular contraction (PVC) morphologies on ECG, including:

1. **PVC 1** – Right bundle branch block (RBBB) configuration with a superior and leftward axis
2. **PVC 2** – RBBB configuration with a narrower QRS complex and superior (rS in inferior leads) and leftward axis
3. **PVC 3** – Left bundle branch block (LBBB) configuration with a very narrow QRS (<90 ms), transition in V3, and leftward axis
4. **PVC 4** – RBBB configuration with an inferior and rightward axis

**Fig. 2:**
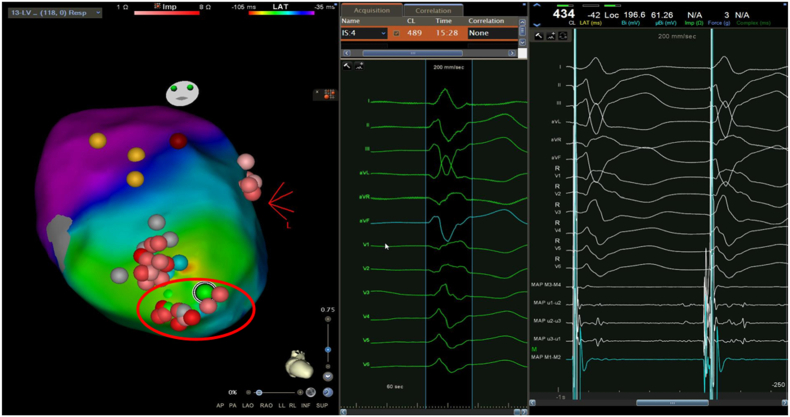
Three-dimensional electroanatomic map and intracardiac electrograms during mapping of the ventricular arrhythmia. **Left Panel:** Red oval showing all lesion sets in the left inferoseptal region. Pace mapping is shown in green, demonstrating a 97% match to the clinical PVC morphology. Red markers represent ablation lesions delivered at sites of earliest activation. **Middle and Right panels:** Corresponding intracardiac electrograms are shown on the right for reference.
• **PVC 2**: Earliest activation (30 ms pre-QRS) mapped to the mid-septum. Ablation resulted in suppression (Fig. 3).

Three-dimensional electroanatomic mapping was performed using the CARTO 3 system and ablation with QDOT catheter (Biosense Webster, Irvine, CA, USA) to reconstruct left ventricular geometry. The following activation sites were identified and targeted:

• **PVC 1**: Pace mapping identified a site with a 97% match to the clinical morphology in the left inferoseptal region, where ablation resulted in successful arrhythmia suppression (Fig. 2).

**Fig. 3:**
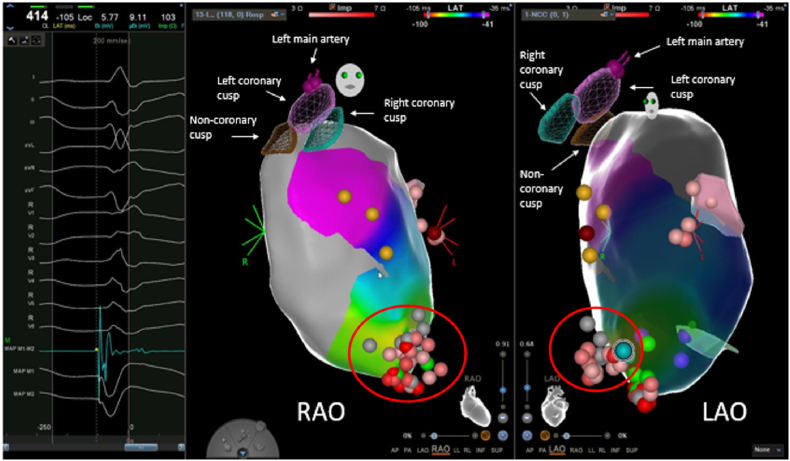
Activation map and anatomic relationship of premature ventricular complex (PVC) origin in the mid septum. **Left panel:** Intracardiac electrograms demonstrating the earliest ventricular activation in the mid septum (highlighted in blue). **Middle and right panels:** Three-dimensional electroanatomic maps (right anterior oblique (RAO) and left anterior oblique projections (LAO), respectively) illustrating the aortic cusps (left, right, and non-coronary) in relation to the left main artery. Red circles showing all lesion sets in the mid septum. The earliest activation site (blue circle with white outline) occurred 30 ms before QRS onset and localized to the left inferoseptal region.
• **PVC 3**: Earliest activation demonstrated a QS complex approximately 1 cm distal to the left bundle potential. Due to proximity to the conduction system and the risk of iatrogenic heart block, ablation was deferred (Fig. 4).

**Fig. 4:**
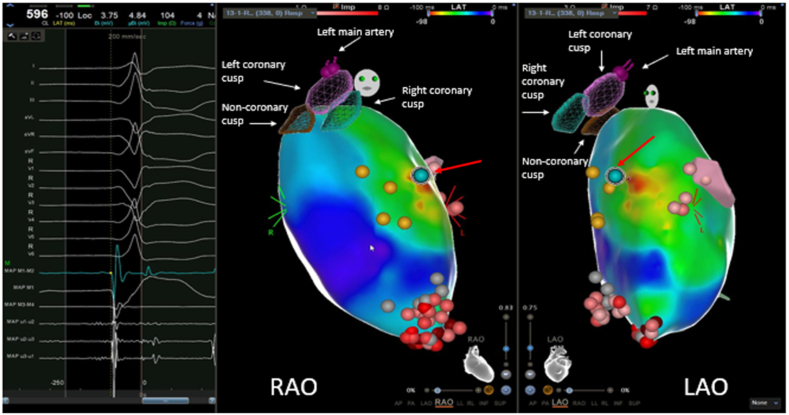
Activation map and anatomic relationship of premature ventricular complex (PVC) origin near the left bundle branch region. **Left panel:** Intracardiac electrograms demonstrating earliest ventricular activation with a QS complex recorded approximately 1 cm distal to the left bundle potential. **Middle and right panels:** Three-dimensional electroanatomic maps (right anterior oblique and left anterior oblique projections, respectively) illustrating the aortic cusps (left, right, and non-coronary) in relation to the left main artery. The earliest activation site (red arrow) occurred 30 ms before QRS onset and localized in close proximity to his left bundle branch (yellow lesions).
• **PVC 4**: Earliest activation (39 ms pre-QRS) localized to the anterolateral papillary muscle (Fig. 5).

**Fig. 5:**
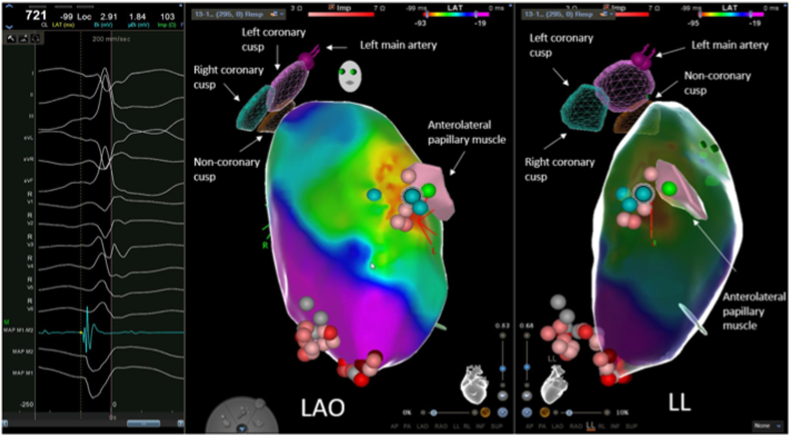
Activation map and anatomic relationship of premature ventricular complex (PVC) origin on the anterolateral papillary muscle. **Left panel:** Intracardiac electrograms demonstrating the earliest ventricular activation at the left coronary cusp (highlighted in blue). **Middle and right panels:** Three-dimensional electroanatomic maps (left anterior oblique (LAO) and left lateral (LL) projections, respectively) illustrating the aortic cusps (left, right, and non-coronary) in relation to the left main artery and anterolateral papillary muscle. The earliest activation site (blue circle with white outline) occurred 39 ms before QRS onset and localized to the anterolateral papillary muscle. The corresponding green lesion denotes the site with the best pace-map correlation (97% match).

No procedural complications occurred.

## 3 Follow up

**Fig. 6:**
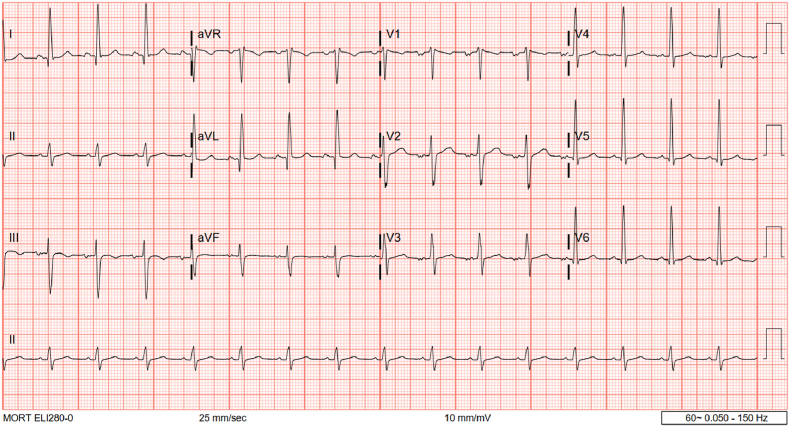
Follow-up 12-lead electrocardiogram demonstrating normal sinus rhythm without premature ventricular contractions following catheter ablation.

Post-procedurally, genetic testing was positive for a heterozygous SCN5A mutation. Given the pre-existing left ventricular dysfunction, the multifocal PVCs (originating from the Purkinje system) were suspected contributors to the reduced ejection fraction. The patient was started on flecainide 50 mg twice daily for residual anteroseptal PVCs, with symptomatic improvement. Due to perceived effects of weakness, the dose was decreased to 25 mg oral twice a day in addition to Metoprolol Succinate 100 mg twice a day. Three-day follow up ECGs was ordered showing normal sinus rhythm (Fig. 6). One month follow-up TTE demonstrated a left ventricular ejection fraction of 40–45% while a 30-day mobile cardiac telemetry demonstrated a decreased in PVC burden to 15%.

## 4 Discussion

This case illustrates a critical diagnostic challenge in contemporary electrophysiology practice: recognizing when ventricle ectopy warrants genetic evaluation. In our patient, severe features prompted earlier suspicion of underlying channelopathy [1]: young age with cardiomyopathy [2], Purkinje-origin PVCs [3], resistance to beta blockers.

This case further highlights the synergistic benefit of combining catheter ablation with class IC antiarrhythmic therapy in the management of a multifocal ectopic Purkinje-related premature contraction (MEPPC)-like phenotype associated with a novel SCN5A variant. In our patient, catheter ablation reduced the predominant PVC foci and arrhythmia burden. However, residual multifocal PVCs—consistent with diffuse Purkinje network involvement—persisted. Subsequent initiation of low-dose flecainide resulted in substantial suppression of residual ectopy and improved ventricular function, reinforcing the concept that sodium channel blockade alone or in combination with ablation could reduce the burden.

MEPPC is increasingly recognized as a distinct clinical entity within the SCN5A channelopathy spectrum. It is characterized by high-burden PVCs arising from the His–Purkinje system and, in some cases, associated left ventricular dysfunction that may be reversible. Ventrella et al. described a similar case involving an SCN5A p.Arg225Gly mutation, where flecainide led to complete arrhythmia suppression and ejection fraction normalization after partial ablation success [8]. Likewise, Keževičiūtė et al. reported dramatic improvements in PVC burden and LVEF with flecainide and mexiletine in a patient with the SCN5A p.Arg814Trp variant after failed ablation attempts [9]. These cases, along with our findings, support a therapeutic paradigm that incorporates sodium-channel blockers to modulate Purkinje hyperexcitability and restore ventricular function.

Mechanistically, gain-of-function mutations in the SCN5A gene enhance late sodium current or impair inactivation kinetics in Nav1.5 channels, predisposing Purkinje fibers to abnormal automaticity and triggered activity. Purkinje fibers are particularly susceptible to these abnormalities due to their unique properties, including more positive resting membrane potentials (−80 to −90 mV), longer action potential durations, and inherent phase 4 automaticity. Enhanced late sodium current in this tissue promotes early afterdepolarizations (EADs) and can trigger multifocal ectopy across the Purkinje network.

Flecainide exerts its antiarrhythmic effects through use-dependent block of cardiac sodium channels, preferentially binding to channels in their inactivated state and thereby suppressing premature depolarizations throughout the Purkinje system-including foci that may not be anatomically accessible or carry high risk of conduction disease with ablation. Catheter ablation, meanwhile, could serve to eliminate dominant ectopic foci and reduce the overall arrhythmogenic burden [10]. This combined approach—targeting both anatomical and molecular substrates—may be especially effective in cases of multifocal Purkinje PVCs.

In our patient, this complementary approach had limited success. Three of four PVC foci were successfully ablated, but a fourth-located approximately 1 cm distal to the left bundle potential-could not be safely targeted. This clinical scenario exemplifies a practical limitation of ablation-only strategy in MEPCC despite younger patients preferring non-pharmacological treatments. Flecainide even at 25 mg twice a day provided partial suppression. The dose was subsequently increase to 50 mg twice a day. While our patient presented predominantly with PVC-related symptoms and cardiomyopathy, the broader arrhythmic implication of SCN5A gain-of-function mutation warrants discussion. Short-coupled Purkinje-triggered PVCs have also been implicated in idiopathic ventricular fibrillation and sudden arrhythmic death syndromes. In such contexts, flecainide and quinidine have been shown to suppress malignant arrhythmogenesis by targeting the Purkinje network [11]. Additionally, SCN5A mutations can manifest with overlapping phenotypes including long QT syndrome type 3, Brugada syndrome, and cardiac conduction disease-each carrying distinct arrhythmia risk profiles [12].

Although our patient did not exhibit high-risk features such as spontaneous ventricular fibrillation, marked QT prolongation, or Brugada ECG pattern sustained ventricular arrhythmias, early initiation of flecainide may have prevented dilated cardiomyopathy. Given the family history of arrhythmia, cascade genetic screening of first-degree relatives. Comprehensive phenotyping including provocative testing for Brugada syndrome and LQTS could be considered to fully characterize arrhythmic risk, and long-term surveillance for emergent high-risk features remains essential.

Class Ic agents, based on the results of the CAST trial, are contraindicated in patients with structural heart disease or cardiomyopathy. Contemporary studies in dilated cardiomyopathy have suggested a potential safe and effective use of class IC agents. SCN5A gain-of-function mutation is a rare disease, based on MRI findings of no delayed enhancement and contemporary evidence it could be reasonable to utilize class IC agents as a first line therapy.

This case underscores the importance of genetic testing in patients with multifocal PVCs and early-onset cardiomyopathy. Identification of SCN5A variants can not only clarify the underlying etiology but also guide therapy, allowing for early initiation of targeted pharmacologic agents. From a clinical perspective, the case supports a hybrid management model that integrates genetic diagnosis, focal ablation, and sodium-channel blockade to achieve durable arrhythmia control.

Key Learning Points:

1. MEPPC is a rare SCN5A channelopathy causing multifocal Purkinje PVCs
2. Sodium channel blockade effective suppresses MEPPC-related ectopy
3. PVC-induced cardiomyopathy could be reversible with arrhythmia suppression
4. Catheter ablation could be an adjunctive therapy but could be restricted due to proximity to the conduction system

## Consent

Written informed consent was obtained from the patient for publication of this case report and accompanying images. Ethical approval was not required for a single case report.

## Ethics statement

The study was conducted in accordance with the principles of the Declaration of Helsinki. Written informed consent was obtained from the patient for publication of this case report and any accompanying images. Ethical approval was not required for this study as it reports a single clinical case.

## Financial disclosure

None.

## Declaration of competing interest

The authors declare that they have no known competing financial interests or personal relationships that could have appeared to influence the work reported in this paper.
